# Correlation Between Ultrasound and Nerve Conduction Study Findings in Evaluating Carpal Tunnel Syndrome

**DOI:** 10.7759/cureus.101588

**Published:** 2026-01-15

**Authors:** Shohel Ahmed, Mohammad Moniruzzaman, Saleh Ahmad, Nusrat M Nipun, Md. Jouel Ahamed, Monia Hafiz, Suriya Shahaly

**Affiliations:** 1 Physical Medicine and Rehabilitation, Ahsania Mission Cancer and General Hospital, Dhaka, BGD; 2 Physical Medicine and Rehabilitation, Dhaka Medical College, Dhaka, BGD; 3 Physical Medicine and Rehabilitation, Holy Family Red Crescent Medical College, Dhaka, BGD; 4 Microbiology, Armed Forces Institute of Pathology, Dhaka, BGD; 5 Physical Medicine and Rehabilitation, Chandpur Sadar Upazila Health Complex, Dhaka, BGD; 6 Physical Medicine and Rehabilitation, Farazy Hospital Ltd., Dhaka, BGD; 7 Physical Medicine and Rehabilitation, Ahsania Mission Medical College, Dhaka, BGD

**Keywords:** bangladesh, carpal tunnel syndrome, cross-sectional area, median nerve, nerve conduction studies, ultrasonography

## Abstract

Background

Carpal tunnel syndrome (CTS) is the most common entrapment neuropathy, caused by median nerve compression within the carpal tunnel, leading to pain, numbness, and functional impairment. Nerve conduction studies (NCS) remain the diagnostic gold standard, while ultrasonography (US) offers a non-invasive complementary modality. This study evaluated the correlation and agreement between US and NCS in CTS.

Methodology

In this cross-sectional study conducted at Dhaka Medical College Hospital from August 2021 to September 2022, 48 symptomatic patients (one hand per patient) underwent standardized NCS (motor distal latency (mDL), amplitude (mAMP), velocity (mCV), sensory distal latency (sDL), amplitude (sAMP), velocity (sCV)) and US at the inlet using a 16-MHz linear probe, with the sonographer blinded to clinical and NCS findings. CTS severity was graded by Bland’s scale (NCS) and cross‑sectional area (CSA) thresholds (US). Spearman correlation assessed associations and Cohen’s kappa quantified inter-modality agreement, with significance set at p-values <0.05.

Results

According to the NCS, 4 (8.3%) patients were normal, 12 (25.0%) were mild, 21 (43.7%) were moderate, and 11 (22.9%) were severe. CSA was positively correlated with mDL (r = 0.667, p < 0.001) and sDL (r = 0.670, p < 0.001) and negatively correlated with sAMP (r = -0.624, p < 0.001) and sCV (r = -0.536, p < 0.001); correlations with mAMP and mCV were not significant. The US and NCS grades were strongly correlated (r = 0.810, p < 0.001). Overall agreement between US and NCS severity was moderate (κ = 0.60, p < 0.001).

Conclusions

US appears to be a practical and reliable adjunct to NCS for evaluating and grading CTS, particularly in resource-limited settings or when NCS are not readily feasible. Larger multicenter studies are needed to further validate its role and refine diagnostic thresholds.

## Introduction

Carpal tunnel syndrome (CTS) arises when the transverse carpal ligament compresses the median nerve, producing pain, numbness, and paresthesia that often radiate beyond its territory; nocturnal symptoms are common, and advanced cases may exhibit thenar muscle weakness [1-3]. It affects about 3.8-7.8% of adults with a pronounced female predominance [4]. Prevalence increases in repetitive‑wrist occupations and among people with pregnancy, diabetes, hypothyroidism, obesity, or menopause [5].

Nerve conduction studies (NCS) are considered the gold standard, but their sensitivity ranges from 49% to 84% with specificity exceeding 95% [6]. The technique is invasive, uncomfortable, and time‑consuming, and has false‑negative rates of 5-10% [5]. High‑resolution ultrasonography (US) has therefore been adopted as a non‑invasive, inexpensive, and rapid complementary modality that visualizes nerve morphology. Various studies report sensitivities between 77% and 94.2% and specificities of 71-98.3% [7,8]. US can aid in the diagnosis and evaluation of CTS as well as differentiate primary from secondary CTS and detect space‑occupying lesions [9].

The cross‑sectional area (CSA) of the median nerve at the carpal tunnel inlet is the most commonly used US metric, yet it depends on age, sex, and body habitus [10]. To compensate for individual variation, derived measures such as the wrist‑to‑forearm ratio (WFR), the difference between distal and proximal CSA, and ratios to the ulnar nerve or carpal tunnel have been proposed [10] and achieved high diagnostic accuracy [10]. Combining CSA with shear‑wave elastography improves detection of mild CTS [11], tailoring cut‑offs to wrist circumference, adopting ΔCSA thresholds around 2.5 mm² [12], and adjusting thresholds in diabetic polyneuropathy [12] further refine diagnostic accuracy. US elastography also quantifies nerve stiffness, which correlates with electrophysiology severity [13]. An optimized wrist circumference‑dependent CSA equation has been proposed to enhance sensitivity over fixed cut‑offs and supports US as a first diagnostic test [14]. Although these advanced sonographic indices have shown promise in previous studies, the present study focused on conventional median nerve CSA measurement, which remains the most widely used and clinically applicable US parameter in routine practice.

Crucially, US findings track electrodiagnostic severity. Median nerve CSA and WFR rise progressively from mild to severe CTS [15] and show moderate positive correlations with sensory and motor latencies and inverse correlations with amplitudes [15]. Despite growing evidence supporting the use of US in CTS, its diagnostic performance relative to NCS has not been adequately evaluated in the Bangladeshi population. Given the invasiveness, discomfort, and limited availability of NCS, it is important to assess whether US correlates reliably with electrodiagnostic findings. Establishing this correlation may support US as an accessible and complementary diagnostic modality in routine clinical practice.

Therefore, the primary objective of this study was to evaluate the correlation between US median nerve CSA and NCS parameters in patients with CTS. In addition, the study aimed to assess the agreement between US and electrodiagnostic severity grading of CTS, to describe the distribution of disease severity based on NCS findings in the study population, and to explore the role of US as a complementary tool to NCS in the evaluation of CTS.

## Materials and methods

Patient selection

**Figure 1:**
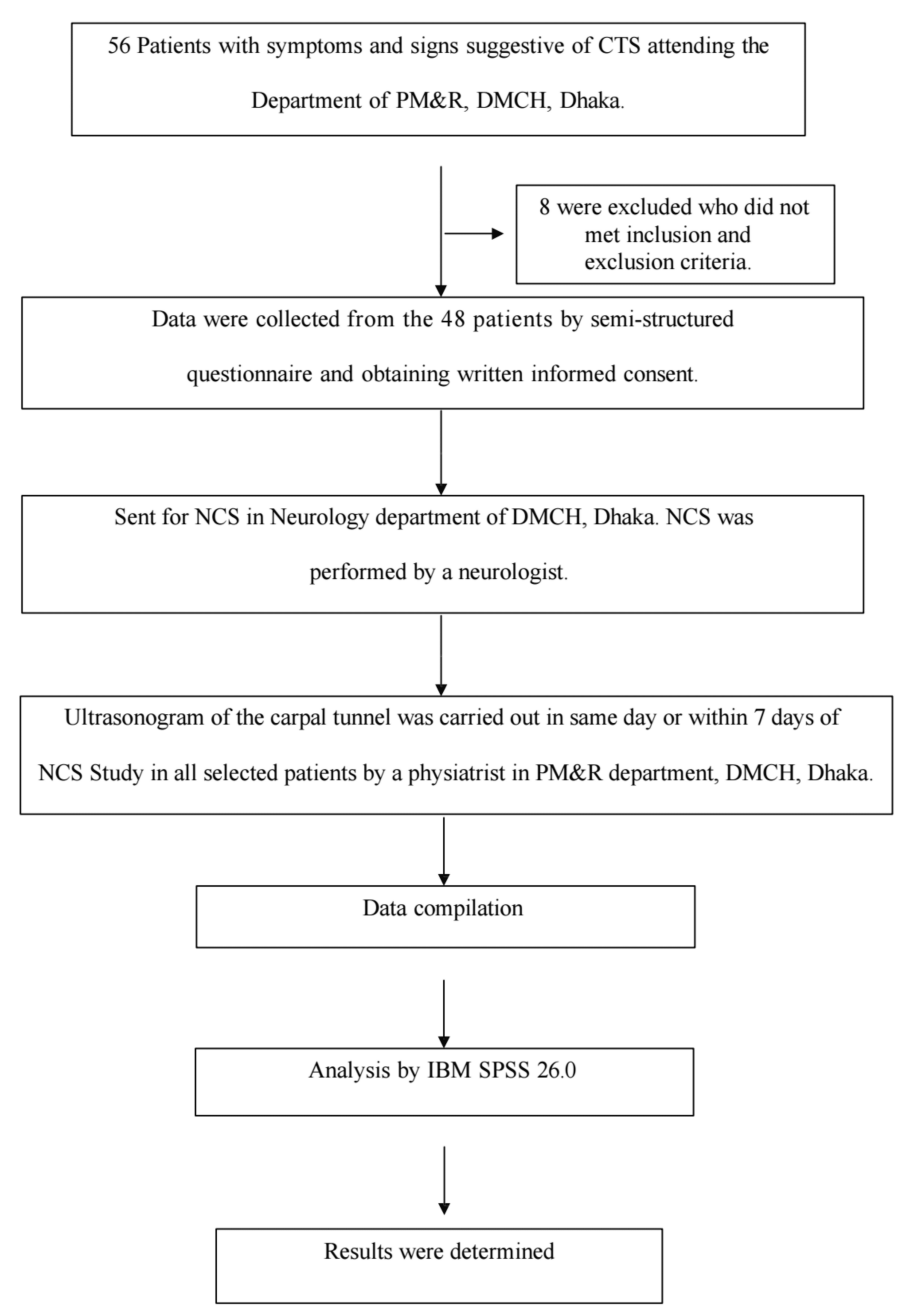
Flow diagram illustrating participant inclusion. CTS = carpal tunnel syndrome; NCS = nerve conduction studies; DMCH = Dhaka Medical College Hospital; PM&R = Physical Medicine and Rehabilitation

This cross-sectional study was conducted from August 1, 2021, to September 30, 2022, at the Department of Physical Medicine and Rehabilitation, Dhaka Medical College Hospital, Dhaka, Bangladesh. A total of 48 patients with clinical suspicion of CTS were recruited. Only the symptomatic hand of each participant was evaluated and included in the analysis (one hand per patient). Inclusion criteria were patients aged 20-60 years of either sex, presenting with symptoms and signs suggestive of CTS, including paresthesia, numbness, pain, weakness, or clumsiness of the hand in the distribution of the median nerve, and positive provocative tests (Phalen’s maneuver and/or Tinel’s sign). Exclusion criteria were patients with systemic peripheral neuropathies (e.g., diabetic polyneuropathy), brachial plexopathy, cervical radiculopathy, rheumatoid arthritis, chronic renal failure, pregnancy, history of wrist fracture or wrist surgery, history of intra-articular steroid injection, or the presence of wrist ganglia (Figure 1).

Sample size calculation

The sample size was estimated for detecting a statistically significant correlation between US median nerve CSA and NCS parameters. The required sample size for testing a correlation coefficient was calculated using Fisher’s z (arctanh) transformation: \[ n = \left( \frac{Z_{\alpha} + Z_{\beta}}{C(r)} \right)^2 + 3 \]

where Zα = 1.96 for a two-sided 5% level of significance, Zβ = 0.84 for 80% power, and \[ C(r) = \frac{1}{2} \ln \left( \frac{1 + r}{1 - r} \right) \]. The anticipated correlation coefficient was taken as r = 0.747 from a previous study reporting a correlation between median nerve CSA and median motor latency [16].

Using Fisher’s transformation:

\[ C(r) = \frac{1}{2} \ln \left( \frac{1 + 0.747}{1 - 0.747} \right) = \frac{1}{2} \ln (6.905) \approx 0.42 \]

Therefore,

\[ n = \left( \frac{1.96 + 0.84}{0.42} \right)^2 + 3 = 44.44 + 3 = 47.44 \]

Thus, the minimum required sample size was 48 participants.

Nerve conduction study

All patients underwent median NCS using a Neuropack S1 (MEB-9400, Nihon Kohden, Japan) performed by a neurologist. Only the symptomatic hand was evaluated and included in the analysis. All NCS were performed in a temperature-controlled room, with hand temperature maintained at ≥32°C to ensure accuracy of latency and conduction velocity measurements. Standard filter settings, sweep speed, and sensitivity were used in accordance with the manufacturer’s recommendations. Standard supramaximal stimulation techniques were applied. For motor studies, stimulation was given above the wrist and at the elbow, and compound motor action potentials (CMAPs) were recorded over the abductor pollicis brevis muscle with surface electrodes. Further, motor distal latency (mDL), amplitude (mAMP), and conduction velocity (mCV) were measured. Electrophysiological parameters were recorded from the best-quality trace, and a single measurement per parameter was used for analysis. For sensory studies, the median nerve was stimulated at the wrist, and recordings were obtained from the second digit with ring electrodes. Sensory distal latency (sDL), sensory amplitude (sAMP), and conduction velocity (sCV) were measured. CTS was graded according to Bland’s neurophysiological scale (Grades 0-6) [17]. For analytical clarity and to facilitate comparison with US grading, Bland’s neurophysiological grades were collapsed into the following four severity categories: normal (grade 0), mild (grades 1-2), moderate (grade 3-4), and severe (grades 5-6) [18]. All NCS were performed by the same experienced neurologist following standardized protocols to ensure consistency and minimize inter-operator variability.

Ultrasonography scanning method and diagnostic protocol

Musculoskeletal ultrasonography of the wrist was performed in the Department of Physical Medicine and Rehabilitation, DMCH, Dhaka, using an SG Healthcare/Q40 ultrasound machine (South Korea) equipped with a 16-MHz linear-array transducer. Subjects were examined in the seated position with forearms supinated and wrists resting on a firm surface. The probe was applied perpendicular to the skin at the level of the carpal tunnel inlet, defined at the level of the pisiform bone. The CSA of the median nerve was measured using the direct tracing method. CTS was graded by CSA as normal (≤10 mm²), mild (>10-13 mm²), moderate (>13-15 mm²), and severe (>15 mm²) [16]. The use of fixed CSA cut-off values was based on their widespread use in prior studies and pragmatic applicability in routine clinical settings. CSA was measured once per wrist to reflect routine clinical practice. Minimal transducer pressure was applied with adequate coupling gel to avoid deformation of the median nerve. All ultrasonographic examinations were conducted by a single trained physiatrist following a standardized scanning protocol, and the examiner was blinded to clinical and NCS findings. Formal intra- or inter-rater reliability analysis was not performed.

Statistical analysis

Data were analyzed using SPSS version 26.0 (IBM Corp., Armonk, NY, USA). Continuous variables were expressed as mean ± standard deviation, while categorical variables were presented as frequency and percentage. Normality of continuous variables was assessed before correlation analysis using the Shapiro-Wilk test. Correlation between NCS parameters and CSA was determined using Spearman’s correlation coefficient due to the non-normal distribution of variables. Agreement between NCS severity and US grading was analyzed using an unweighted Cohen’s kappa statistic. A p-value <0.05 was considered statistically significant, and there were no missing data in the dataset.

## Results

Participant characteristics

In total, 48 clinically suspected CTS hands were analyzed. The cohort was predominantly female (46, 95.8%), with a mean age of 40.4 ± 9.4 years (range = 25-60). Most participants were housewives (40, 83.3%). The mean body mass index (BMI) was 21.5 ± 2.6 kg/m² (range = 18-28), and CTS most often involved the right hand (41, 85.4%). The average symptom duration was 16.5 ± 11.4 months (range = 3-60), with one-third reporting 13-24 months of symptoms (Table 1).

**Table 1:** Distribution of sociodemographic and clinical characteristics of participants (n = 48). Data are presented as frequency and percentage.

Variables	Frequency	Percentage (%)
Age (years)		
25–34	13	27.1
35–44	17	35.4
45–54	12	25.0
≥55	6	12.5
Mean ± SD (range)	40.4 ± 9.4 (25–60)	
Sex		
Male	2	4.2
Female	46	95.8
Occupation		
Housewife	40	83.3
Service holder	3	6.3
Businessman	2	4.2
Day laborer	2	4.2
Student	1	2.0
Body mass index (kg/m²)		
Underweight	4	8.3
Normal	38	79.2
Overweight	6	12.5
Mean ± SD (range)	21.5 ± 2.6 (18–28)	
Hand involved		
Right	41	85.4
Left	7	14.6
Duration of symptoms (months)		
≤6	13	27.1
7–12	13	27.1
13–24	16	33.3
≥24	6	12.5
Mean ± SD (range)	16.5 ± 11.4 (3–60)	

Electrodiagnostic severity distribution

Based on the collapsed NCS grade, 4 (8.3%) patients were classified as normal, 12 (25.0%) as mild, 21 (43.7%) as moderate, and 11 (22.9%) as severe (Table 2).

**Table 2:** Carpal tunnel syndrome severity based on nerve conduction study findings (n = 48). Data are presented as frequencies and percentages.

Severity (collapsed)	Frequency	Percentage (%)
Normal	4	8.3
Mild	12	25.0
Moderate	21	43.7
Severe	11	22.9

Correlation between NCS parameters and median nerve CSA

Spearman correlation demonstrated strong positive correlations between ultrasound CSA and distal latencies (motor mDL: r = 0.667, p < 0.001; sensory sDL: r = 0.670, p < 0.001). CSA was negatively correlated with sensory indices (sAMP: r = -0.624, p < 0.001; sCV: r = -0.536, p < 0.001). The correlations with motor conduction measures did not reach significance (mAMP: r = -0.204, p = 0.190; mCV: r = -0.246, p = 0.112) (Table 3).

**Table 3:** Correlation between nerve conduction study parameters and cross-sectional area (n = 48). Spearman correlation was done; *: p-value <0.05 was considered statistically significant; **: p-value <0.001 was considered statistically significant.

Nerve conduction study parameters	Spearman’s r	P-value
Motor distal latency (mDL)	0.667	<0.001**
Motor amplitude (mAMP)	–0.204	0.190
Motor conduction velocity (mCV)	–0.246	0.112
Sensory distal latency (sDL)	0.670	<0.001**
Sensory amplitude (sAMP)	–0.624	<0.001**
Sensory conduction velocity (sCV)	–0.536	<0.001**

Correlation between the grading of CTS according to US and CTS

Table 4 shows that there was a significant positive correlation between the grading of CTS according to US and the grading of CTS according to NCS, with a Spearman correlation coefficient (r) of 0.810 (p < 0.001).

**Table 4:** Correlations between grading of carpal tunnel syndrome (CTS) according to ultrasound (US) and nerve conduction study (NCS) (n = 48). Spearman correlation was performed; *: p-value <0.05 was considered statistically significant; **: p-value <0.001 was considered statistically significant.

Criteria	Grading of CTS according to US	P-value
Grading of CTS according to NCS	0.810	<0.001**

Agreement between US and electrodiagnostic severity grading

As shown in Table 5, the result of the test using the kappa statistic was 0.60, indicating that there is a moderate match between the US and NCS examination results.

**Table 5:** Agreement between nerve conduction study (NCS) and ultrasound (US) in carpal tunnel syndrome severity. *: Cohen’s kappa = 0.60; p < 0.001.

Severity of CTS NCS	Severity of CTS according to US				
	Normal	Mild	Moderate	Severe	Total
Normal	0	4	0	0	4
Mild	0	8	4	0	12
Moderate	0	4	16	1	21
Severe	0	0	0	11	11
Total	0	16	20	12	48

## Discussion

This study enrolled 48 hands from symptomatic individuals with clinical suspicion of CTS. The cohort was almost entirely female (95.8%) with a mean age of about 40 years, and most participants were housewives with normal BMI. CTS was more common in the right hand (85%) and had been present for a mean of 16.5 months, indicating chronicity. These demographic findings mirror the epidemiology of CTS reported in population‑based studies. The disorder is significantly more common in women, especially between the fourth and sixth decades of life; prevalence estimates in general populations range 3.8-5%, with peak incidence around 40-60 years [19,20]. Several factors may explain the female preponderance, including a narrower carpal tunnel, hormonal influences on connective tissue and synovial swelling during pregnancy, and higher exposure to repetitive domestic chores [21]. Occupational stress and repetitive wrist movements are also important; our predominance of housewives may reflect repetitive household tasks and unmeasured socioeconomic factors [19]. The normal BMI observed in most participants contrasts with other studies where obesity and metabolic syndrome were significant risk factors. This difference could arise from cultural dietary habits in our setting or the selection of predominantly non‑obese individuals.

Our cohort’s mean age and symptom duration were similar to those reported in recent Asian studies that examined US and NCS in CTS. For example, Emril et al. studied 46 patients in Indonesia with a mean age of 44 years and noted that women constituted 80.4% of the sample [22]. In Turkey, Yurdakul et al. observed that CTS occurs most often in middle‑aged women and reported an average age of around 46 years [23]. These similarities suggest that our participants represent typical CTS patients in low‑ and middle‑income countries. Slight differences in age distribution may be attributed to varying inclusion criteria, with some series focusing on post‑menopausal women or including older patients with comorbidities such as diabetes or hypothyroidism, both known to increase CTS risk [21].

Electrophysiological grading by NCS showed that 8.3% of hands were normal, while 25% were mild, 43.7% moderate, and 22.9% severe CTS. The predominance of moderate-to-severe cases likely reflects the referral‑based nature of our cohort; patients often seek specialist care only after prolonged symptoms. A study noted that the majority of participants had mild CTS (59.2%), with a mean age of 52 years, and emphasized that mild cases are often detected during screening rather than symptomatic referrals [24]. Taken together, the spectrum of electrodiagnostic severity in our series underscores that a substantial proportion of patients present with advanced disease.

We observed strong positive correlations between median nerve CSA measured at the carpal tunnel inlet and distal latencies on NCS. Conversely, CSA showed significant negative correlations with sAMP (r = -0.624) and sCV (r = -0.536), whereas correlations with mAMP or mCV were not significant. These findings indicate that larger nerve swelling is associated with slowed conduction and reduced sensory responses, reflecting demyelination and early axonal compromise. Comparable patterns have been documented in multiple studies [23,25,26]. Discrepancies among studies may result from differences in US technique (inlet vs tunnel mid‑portion), patient positioning, and definitions of electrophysiological parameters.

When CTS severity was categorized by US grading using CSA thresholds and by NCS grading, Spearman’s correlation coefficient was 0.810 (p < 0.001), indicating a strong positive relationship between structural and functional severity scales. This suggests that increasing nerve swelling on US parallels electrophysiological worsening and that sonographic grading provides a valid proxy for NCS severity. Similar concordance has been reported in previous studies [22,24,27]. Our correlation coefficient surpasses those reported in these studies, possibly reflecting a consistent US technique or narrower variability in CSA due to our homogeneous population. Nevertheless, the strong correlation across studies reinforces the principle that sonographic CSA increases in tandem with electrophysiological impairment.

Despite the strong correlation between ultrasound and NCS grades, the kappa statistic measuring agreement between the two classification systems in our study was 0.60, signifying moderate concordance. This moderate Cohen’s kappa value indicates meaningful agreement between US and NCS in grading CTS. Clinically, this suggests that US can reliably complement NCS in routine practice, particularly when electrophysiological testing is unavailable or poorly tolerated. Several studies support this finding [22,24,27,28]. These values, similar to ours, fall in the moderate to substantial agreement range. Our moderate kappa suggests that while US and NCS often concur, they are not interchangeable; each modality may identify certain cases that the other misses. Several factors may contribute to discordant classifications. US captures morphological changes that may precede electrophysiological abnormalities; early nerve swelling due to edema or synovial hypertrophy can yield enlarged CSA despite normal latencies and velocities [21]. Conversely, NCS may detect conduction slowing in cases with minimal swelling, particularly when demyelination occurs without marked edema. Differences in measurement site (proximal inlet vs. mid‑tunnel) and US machine resolution can also influence CSA estimates. Moreover, comorbid conditions such as diabetes may reduce nerve conduction velocity independent of carpal tunnel compression, leading to a higher NCS grade without commensurate swelling.

The present study has several limitations. This was a cross-sectional, single-center study with a relatively small, purposively selected sample (48 hands; one hand per patient), which limits causal inference and generalizability. The study population was predominantly female, reflecting the known epidemiology of CTS; however, this sex distribution may limit extrapolation of the findings to male populations. US grading was based on fixed CSA cut-offs without wrist-size adjustment, and inter- or intra-rater reliability, as well as complementary sonographic metrics (ΔCSA, WFR, elastography) were not assessed. NCS served as the reference standard rather than surgical or longitudinal clinical outcomes, and the observed moderate agreement (κ = 0.60) indicates partial discordance between structural and electrophysiological assessments. Future multicenter prospective studies with larger and more diverse samples and standardized sonographic protocols are warranted.

## Conclusions

The present study demonstrates a strong correlation between US median nerve CSA and NCS parameters, along with moderate agreement in electrodiagnostic severity grading. These findings support US as a practical, non-invasive adjunct for the evaluation and grading of CTS in day-to-day clinical practice, particularly in resource-limited settings such as Bangladesh, where access to NCS may be restricted or poorly tolerated. However, the conclusions should be interpreted in light of the study’s relatively small sample size. Larger, multicenter studies using standardized cut-offs and incorporating advanced sonographic indices are warranted to further validate US as a frontline diagnostic modality.
